# Molecular heterogeneity of β‐thalassemia variants in the Eastern region of Morocco

**DOI:** 10.1002/mgg3.1970

**Published:** 2022-05-26

**Authors:** Ihab Belmokhtar, Saida Lhousni, Mounia Elidrissi Errahhali, Ayad Ghanam, Manal Elidrissi Errahhali, Zaina Sidqi, Meryem Ouarzane, Majida Charif, Mohammed Bellaoui, Redouane Boulouiz, Noufissa Benajiba

**Affiliations:** ^1^ Genetics Unit, Medical Sciences Research Laboratory, Faculty of Medicine and Pharmacy University Mohammed Premier Oujda Morocco; ^2^ Department of Pediatrics Mohammed VI University Hospital, Faculty of Medicine and Pharmacy, University Mohammed Premier Oujda Morocco; ^3^ Transfusion Regional Centre Oujda Morocco

**Keywords:** BRO Biobank, hemoglobin, molecular profile, morocco, variant, β‐thalassemia

## Abstract

**Background:**

β‐thalassemia syndromes are the most common hereditary blood disorders in the world and are recognized as a major health problem in Morocco. They are characterized by the reduction or the absence of β‐globin chain synthesis. The severity of the disease depends on the nature of the variants affecting the β‐globin gene (*HBB*), and each ethnic group has its own mutation spectrum. Hereby, we present, for the first time, the molecular profile of β‐thalassemia in the Eastern region of Morocco.

**Methods:**

This study concerns 39 cases from 33 families who were enrolled in the BRO Biobank. Nineteen were diagnosed with β‐thalassemia major and 20 with β‐thalassemia minor. To detect mutations of the β‐globin gene, we have used RFLP‐PCR and Sanger sequencing.

**Results:**

Nine known β‐thalassemia variants have been identified. Among these, we reported, for the first time in the Moroccan population, the Czechoslovakian variant C38/39(‐C) at homozygous state. The C39(C > T) was the most frequent variant (72.54%), followed by FSC5(‐CT) (5.88%), FSC6(−A), IVS‐1‐110(G > A), −29(A > G), C38/39(‐C) (3.92% each), and finally by IVS‐I‐1(G > A), IVS‐II‐1(G > A), and −56(G > C) (1.96%). Of particular interest this mutational spectrum of β‐thalassemia is very different from that found in previous studies in Morocco or in other North African countries.

**Conclusion:**

This study is the first contribution to the description of the molecular profile of β‐thalassemia in the Eastern region of Morocco. It shows the high molecular heterogeneity of β‐thalassemia in our country. Therefore, these results can be valuable for the implementation of carrier screening, genetic counseling, and prenatal diagnosis programs.

## Graphical abstract

The molecular investigation of β‐thalassemia in eastern region of Morocco indicates a high molecular heterogeneity and unique variant spectrum compared to that reported in other region of Morocco or in other North African countries. These data can be valuable for the implementation of carrier screening, genetic counseling and prenatal diagnosis programs.

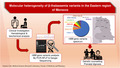

## 1 INTRODUCTION

β‐thalassemia syndromes are the most prevalent hereditary blood disorders worldwide characterized by the reduction (β^+^) or the absence (β^0^) of β‐globin chain synthesis, causing hypochromic, microcytic anemia hemolysis, and erythropoiesis defect. It includes three main forms: thalassemia major, thalassemia intermedia, and thalassemia minor. β‐thalassemia can also be associated with other structural hemoglobin variant such as sickle cell disease (HbS), hemoglobin C (HbC), hemoglobin E (HbE), (Hassan et al., 2016; Weatherall & Clegg, 2008).

Clinically, individuals with thalassemia major show a mucocutaneous pallor, severe anemia, hepatosplenomegaly, and therefore, require regular transfusions. Patients with thalassemia intermedia are diagnosed later at the age of 2 to 6 years old with a milder anemia or in adulthood with bones and face deformities and gallstones; they usually do not require a regular transfusion. Individuals with thalassemia minor, also known as carriers, are asymptomatic and have slight anemia (Cappellini et al., 2008; Olivieri, 1999).

The β‐thalassemia is originally common in tropics and subtropics areas such as the Mediterranean countries, the Middle East, central Asia, India, and Southern China as well as countries along the north coast of Africa and in South America. Nowadays, β‐thalassemia is the most common disease all over the world as a result of population migration (Weatherall & Clegg, 2001).

Several molecular studies have identified a large number of variants in the β‐globin gene *HBB* (MIM141900). Most of them are single base pair mutations in functionally important regions, and have been reported in the Human Hemoglobin Variants Database (see Globin Gene Server at website: http://globin.cse.psu.edu/). The high prevalence of certain variants in specific populations has greatly facilitated molecular diagnosis and genetic counseling. Thus, the common widespread variant in Lebanon, Egypt, Syria, Jordan, Tunisia, and Algeria is the IVS‐1‐110(G > A) (Başak, 2007; El‐Hazmi et al., 1995; Hamamy & Al‐Allawi, 2013; Makhoul et al., 2005). While, in the Eastern Arabian Peninsula and the Asian Indian, the variants (IVS‐I‐5(G > C), C8/9(+G), and IVS‐I(−25bpdel)) were more common (Baysal, 2005; Panigrahi & Marwaha, 2007).

As in many Mediterranean countries, thalassemia is a major public health concern in Morocco and has not been fully characterized. The previous studies on the genetic basis of β‐thalassemia were mainly focused in the western and northern areas and have revealed high molecular defects heterogeneity (Agouti et al., 2007; Agouti, Badens, et al., 2008; Lemsaddek et al., 2003). However, there is no data about the molecular profile of β‐thalassemia in the eastern region of Morocco. Therefore, the aim of this study is to investigate the molecular defects in patients with β‐thalassemia in this region which is important for carrier screening, and for establishing genetic counseling and prenatal diagnosis in Morocco.

## 2 MATERIAL AND METHOD

### 2.1 Description of the study and ethics statement

This cohort study was achieved in the Genetics Unit of the Faculty of Medicine and Pharmacy of Oujda. According to the BRO Biobank protocol (Lhousni et al., 2020), the participants were enrolled by the Pediatric departments of the University Mohammed IV Hospital of Oujda and the Regional Center of Blood Transfusion of Oujda, Morocco.

The study was approved by the ethical committee, of the Faculty of Medicine and Pharmacy of Casablanca, in accordance with the declaration of Helsinki standard.

### 2.2 Subjects

The study was conducted on 29 individuals from the BRO Biobank. Nineteen patients from 18 families were diagnosed with β‐thalassemia major and 20 subjects from 15 families have β‐thalassemia minor. All the subjects or their representative family gave written informed consent to the study.

### 2.3 Mutation screening

DNA extraction from whole blood and saliva sample was performed manually by the phenol chloroform‐isoamyl alcohol method. The most common β‐thalassemia variants in the Moroccan population (C39(C > T), IVSI‐1(G > A) and FSC6(−A)) were characterized using PCR followed by restriction fragment length polymorphism (RFLP). The primers used for amplification of *HBB* gene region (HBB‐mut) were previously described (Clark & Thein, 2004). The PCR products were directly digested by BfaI, Bsu36I, and BsaBI Restriction enzymes (New England Biolabs, Inc.) to check the three most common variants, respectively (C39(C > T), IVSI‐1(G > A), and FSC6(−A)).

In order to confirm the PCR‐RFLP results and to look for other variants in the *HBB* gene, two regions were sequenced (Seq1 and seq2). Sanger sequencing was performed on an ABI 3500 Genetic Analyzer (Applied Biosystems‐Thermofisher). The sequences were compared to the *HBB* reference sequence (NM_000518.5) using the SeqScape Software Version 4.0 (Applied Biosystems).

### 2.4 Statistical analysis

Statistical analysis was carried out using IBM SPSS Statistic Software Version 21. A *p*‐value < .05 was considered statistically significant.

## 3 RESULTS

### 3.1 Clinical and biological characteristics of the participants

Among the 39 cases, 26 cases (66.67%) were female and 13 (33.33%) were males (sex‐ratio F/M: 2.02). The sex‐ratio F/M was 1.77 and 2.33 for β‐thalassemia major and β‐thalassemia minor, respectively. No statistically significant differences were observed for sex‐ratio (*p* = .651). The consanguinity was estimated at 38.89% in patients with β‐thalassemia major and 40% for those with β‐thalassemia minor, and subsequently the mean consanguinity rate was estimated at 39.39%. The majority of the recruited families were from Oujda‐Angad region (46.15%), followed by Nador (25.64%), Berkane (12.82%), Al Hoceima (10.26%), Driouch (2.56%), and Guercif (2.56%).

The median age at diagnosis was around 6 months for patients with β‐thalassemia major and 3‐years‐old for those with β‐thalassemia minor. β‐thalassemia is clinically heterogeneous, all Patients with β‐thalassemia major have developed more serious symptoms while β‐thalassemia trait patients showed mild symptoms. Table 1.summarizes the clinical features and hematological parameters of the participants.

**TABLE 1:** Distribution of clinical features and some hematological parameters

	Major β‐thalassemia		Minor β‐thalassemia		All patients	
	nb	%	nb	%	nb	%
Gender						
Male	7	36.84%	6	30.00%	13	33.33%
Female	12	63.16%	14	70.00%	26	66.67%
Sex‐Ratio F/M	1.71		2.33		2.02	
Provenance						
Oujda	5	26.32%	13	65.00%	18	46.15%
Nador	6	31.58%	4	20.00%	10	25.64%
Al Hoceima	3	15.79%	1	5.00%	4	10.26%
Berkane	3	15.79%	2	10.00%	5	12.82%
Driouch	1	5.26%	0	0%	1	2.56%
Guercif	1	5.26%	0	0%	1	2.56%
Symptoms						
Mucocutaneous Pallor	19	100%	13	65.00%	–	
Hepatosplenomegaly	10	52.63%	0	0%	–	
Jaundice	5	26.32%	2	10.00%	–	
Asthenia	1	5.26%	4	20.00%	–	
Mean age of diagnosis (months)	10.2 ± 13.47 [1–48]		33.23 ± 16.36 [12–60]			
Median	6		36			
Hematological data^a^						
Hb (g/dl)	6.88 ± 1.42 [4.7–9.6]		9.93 ± 1.01 [7.6–11.1]		–	
MCV (fl)	74.78 ± 6.2 [60.5–82.6]		58.21 ± 1.91 [55–61.3]		–	
MCH (pg)	25.16 ± 2.56 [19–27.4]		17.98 ± 1.19 [15.8–19.7]		–	
MCHC (g/dl)	33.37 ± 1.73 [30.7–36.4]		30.66 ± 1.69 [27.9–33.1]		–	
HbA1 (%)	3.41 ± 4.78 [0–11.4]		89.71 ± 3.37 [81–93.1]		–	
HbA2 (%)	2.13 ± 0.88 [1,6–3.9]		5.28 ± 0.79 [3.8–6.1]		–	
HbF (%)	94.45 ± 5.16 [86.9–98.4]		5.01 ± 3.91 [0.9–15]		–	

Abbreviations: ALL, acute lymphoblastic leukemia; Hb, hemoglobin; HbA1, hemoglobin A1; HbA2, hemoglobin A2; HbF: fetal hemoglobin; MCH, mean corpuscular hemoglobin; MCHC, mean corpuscular hemoglobin concentration; MCV, mean corpuscular volume.

^a^ The subjects with compound heterozygous β‐thal/sickle and β‐thal/ALL were excluded from CBC and CE the analysis.

Among the participants, one subject was compound heterozygous for HbS and β‐thalassemia, and had a skin pallor and fatigue due to severe microcytic hypochromic anemia (Hb: 5.9 g/dl, MCV: 57 fl, and MCH: 17.89 pg). Hemoglobin electrophoresis showed the presence of HbS and elevated rate of HbA2 and HbF (HbA1: 0%, HbA2: 4.9%, HbF: 20.1%, and HbS: 75%).

Another participant was diagnosed with β‐thalassemia minor and Acute Lymphoblastic Leukemia (ALL) at the age of 2 years old. He had skin pallor, bone pain, and fatigue due to the severe anemia (Hb: 5.4 g/dl, MCV: 50 fl, and MCH: 16.7 pg). Hemoglobin electrophoresis showed a β‐thalassemia trait profile (HbA1:92.5%, HbA2: 5%, and HbF: 2.5%).

### 3.2 Molecular analysis

Thirty‐nine patients were studied, 19 with β‐thalassemia major and 20 with β‐thalassemia minor. As six patients were found in sibling, they were excluded when calculating the frequency of β‐globin gene variants. Therefore, 52 mutated alleles were evaluated. A total of 10 β‐globin gene variants were detected; most of them have a Mediterranean origin. According to their effect on gene function, five variants affect the secondary structure of the β‐globin protein such as C39(C > T), FSC6(−A), FSC5(‐CT), C38/39(‐C), and SCD6(A > T), three variants lead to defective mRNA synthesis such as IVS‐I‐1(G > A), IVS‐1‐110(G > A), and IVS‐II‐1(G > A). The last two variants (−29(A > G), −56 (G > C)) are in the promoter region, and decreases the abundance of the β‐globin mRNA.

The allelic frequency distribution showed that the C39(C > T) is the most frequent variant (71.15%), followed respectively by FSC5(‐CT) (5.77%), FSC6(−A), IVS‐1‐110(G > A), −29(A > G), and C38/39(‐C) 3.85% for each and finally by IVS‐I‐1(G > A), IVS‐II‐1(G > A), −56(G > C), and SCD6(A > T). Surprisingly, the C38/39(‐C) variant has never been reported before in Morocco. Our patient was homozygous, while the parents and his sibling were heterozygous for this variant.

Concerning the genotypic pattern, among the 18 patients suffering from β‐thalassemia major, 12 (67%) were homozygous while six patients (33%) were compound heterozygous. Table 2 shows a summary of the genotypic pattern of the studied families. The frequency of homozygous variants was 55.56% for C39(C > T) and 5.56% for FSC5(‐CT) and C38/39(‐C). It should be noted that all compound heterozygous patients carried the C39 in one allele. While the second allele was IVS‐I‐110(G > A) in two cases and − 29(A > G), IVS‐II‐1(G > A), FSC5(‐CT), or SCD6(A > T) in only one case each.

**TABLE 2:** Genotypic pattern in population study

Genotypes	*n*	%
β‐thalassemia major		
C39(C > T)/C39(C > T)	10	55.56%
C39(C > T)/IVS‐I‐110(G > A)	2	11.11%
C39(C > T)/−29(A > G)	1	5.56%
C39(C > T)/IVS‐II‐1(G > A)	1	5.56%
C39(C > T)/FSC5(‐CT)	1	5.56%
C39(C > T)/SCD6(A > T)	1	5.56%
FSC5(‐CT)/FSC5(‐CT)	1	5.56%
C38/39(‐C)/C38/39(‐C)	1	5.56%
β‐thalassemia minor		
C39(C > T) /Wt	11	71.33%
FSC6(−A) /Wt	2	13.33%
IVS‐I‐1(G > A) /Wt	1	6.67%
−29(A > G)/−56(G > C)	1	6.67%

On the other hand, we have identified four intragenic polymorphisms (C2(T > C) (rs713040), IVS‐II‐16(G > C) (rs10768683), IVS‐II‐74(T > G) (rs7480526), and IVS‐II‐666(C > T) (rs1609812)). The combination of these polymorphisms defines three different frameworks **(**Table 3). The most frequent framework is the FWI (CCGT) (71.88%), followed by FWII (CCTT) (25.00%) and then FWIII (TGTC) (3.12%). The distribution of the pathogenic variant compared to the frameworks shows that C39(C > T), −29(A > G), IVS‐I‐110(G > A), −56(G > C) variants were associated exclusively with FWI, while FSC5(‐CT), IVS‐II‐1(G > A), IVS‐I‐1(G > A), and SCD6(A > T) variants were associated exclusively with FWII. However, FSC6(−A) was found to be associated with FWI in one family and with FWII in other family. Interestingly, the C38/39(‐C) variant, which has never been reported before in the Moroccan population, was solely associated with FWIII (Table 3).

**TABLE 3:** Distribution of HBB frameworks and their association with the pathogenic variants

Frameworks	β‐thalassemia major		β‐thalassemia minor		Total		Associated variants
FW I (CCGT)	29	80.56%	13	86.67%	42	71.88%	−56(G > C), −29(A > G), IVS‐I‐110(G > A), C39(C > T), FSC6(−A)
FW II (CCTT)	5	13.89%	2	13.33%	7	25.00%	FSC5(‐CT), FSC6(−A), SCD6(A > T), IVS‐I‐1(G > A), IVS‐II‐1(G > A)
FW III (TGTC)	2	5.56%	0	0%	2	3.12%	C38/39(‐C)
nb of chr	36	100%	15	100%	51	100%	

Moreover, two other polymorphisms at the 3′UTR region of the β‐globin gene, (3′UTR + 34(C > A) (rs1277208445) and 3′UTR + 101(G > C) (rs12788013)), were identified at heterozygous state in the patient with β‐thalassemia minor and ALL.

## 4 DISCUSSION

β‐thalassemia is a blood disorder that result in inherited defects in the hemoglobin β‐globin chains production. It is the most common genetic disorder worldwide and is recognized as a major public health concern in the Mediterranean countries, the middle‐Eastern, Indian regions, and Southeast Asia and particularly in North Africa (Agouzal et al., 2010). The incidence and prevalence rates of thalassemia are influenced by demographic and cultural characteristics of populations. For instance, it is well documented that Morocco and all Arabic countries display high rates of consanguinity (Mohammed et al., 2019; Tadmouri et al., 2009; Talbi et al., 2007). In this study, the consanguinity rate among the β‐thalassemia patients was estimated at 39.39%. This finding is consistent with the previous study which showed that the consanguinity rate in Eastern Morocco region is higher than the national average (29.58% vs. 22.79%) (Mohammed et al., 2019; Talbi et al., 2007). Thereby, it is not surprising to see a strong occurrence of autosomal recessive diseases such as thalassemia in our population (Habibeddine et al., 2018; Jaouad et al., 2009; Laghmich et al., 2019).

Regarding molecular investigation of β globin gene, we report here, nine β‐thalassemia pathogenic variants (C39(C > T), FSC6(−A), IVS‐I‐1(G > A), −29(A > G), FSC5(‐CT), IVS‐1‐110(G > A), C38/39(‐C), IVS‐II‐1(G > A), and −56 (G > C)), and one sickle cells allele SCD6(A > T). Beside, four intragenic polymorphisms (C2(T > C), IVS‐II‐16(G > C), IVS‐II‐74(T > G), and IVS‐II‐666(C > T)) have been identified, which define three Frameworks. Framework identification could be used as an indirect method in prenatal diagnostic programs to track variant alleles and thus differentiate between normal and mutant alleles (Akhavan‐Niaki et al., 2012; Hashemi‐Soteh et al., 2017). Furthermore, two other polymorphisms at the UTR region (3′UTR + 34(C > A) and 3′UTR + 101(G > C)) were found. The 3′UTR + 101(G > C) was reported only in Palestinian β‐thalassemia patients (Sirdah et al., 2013) while the 3′UTR + 34 (C > A) variant have not been investigated yet and was reported in ClinVar database as likely benign SNV contrary of the 3′UTR + 101(G > C) (Smith et al., 2015). Altogether, this variant profile confirms the high molecular heterogeneity and could explain the frequency (33%) of compound heterozygosity described in our study.

It is to highlight that all of the variants were previously described in the Moroccan population except for the C38/39(‐C) which has never been reported before. The patient, originated from Guercif, was homozygous, while the parents and the siblings were carriers. The C38/39(‐C) is very rare variant and was only found in Czechoslovakian population and most affected families come from central Moravia (Divoka et al., 2016; Indrak et al., 1991, 1992). Previous haplotype analysis studies showed that C38/39(‐C) is linked only to Haplotype II (HindIII Gγ; HinfIβ; HincII β ψ; RsaI β; AvaII β; HindIII Aγ [− + − − − +]) (Kynclová et al., 1998) and interestingly in consistent with this data, β^0^C38/39(‐C) was the one and only variant linked to the FWIII in our study, indicating a distinct origin or a recent occurrence. Further studies are necessary to understand the occurrence of this variant in the eastern region of Morocco.

Concerning the distribution of variants frequencies between several studies conducted in Moroccan population, it seems obvious that the mutational spectrum of β‐thalassemia found here is very different from that found in previous studies in Morocco or in other North African countries (Agouti et al., 2007; Agouti, Badens, et al., 2008; Lemsaddek et al., 2003, 2004). Two major differences were observed: First, all the β‐thalassemia major patients were homozygous or compound heterozygous for C39(C > T). Furthermore, it has been noticed that this variant, though the most common in all previous studies, showed a much higher frequency (71.15%) compared to those found in Morocco (15.56%–28.05%) (Agouti et al., 2007; Agouti, Badens, et al., 2008; Lemsaddek et al., 2003, 2004), in Algeria (26%–43%) (Abdaoui et al., 2019; Bennani et al., 1994; Boudrahem‐Addour et al., 2009; Labie et al., 1990) and in Tunisia (49%–37.50%) (Chouk et al., 2004; Fattoum et al., 2004; Sahli et al., 2016). The second particularity in our result concern the three variants (FSC8(−AA), IVS‐I‐6(T > C), and IVS‐II‐745(C > G)) described in previous studies as three of the five most common variants in Morocco, are surprisingly absent in the Eastern region of Morocco and are rare in Algerian and Tunisian population. Table 4 shows the significance variation of the variants found in our study compared to the previous studies in Morocco and the last two published studies in Algeria and Tunisia.

**TABLE 4:** Comparison of the distribution of variants frequencies with the previous studies in Morocco, Algeria, and Tunisia

Variants	Present study	Morocco					Algeria			Tunisia		
		a	b	c	d	*p*‐Value	e	f	*p*‐Value	g	h	*p*‐Value
C39(C > T)	71.15%	26.20%	15.60%	28%	26.60%	**.0001**	26.00%	43.00%	**.0001**	49.00%	37.50%	**.0001**
FSC5(‐CT)	5.77%	0%	0%	0%	1.30%	**.003**	0%	0%	–	040%	0%	**.015**
FSC6(−A)	3.85%	13.40%	10%	9.80%	5.70%	**.092**	13.00%	6.70%	**.006**	2.60%	2.08%	**.0001**
−29(A > G)	3.85%	4.30%	6.70%	8.50%	6.30%	**.647**	1.40%	3.33%	**.03**	0%	0%	**.011**
IVS‐I‐110(G > A)	3.85%	3.20%	2.20%	0%	5.70%	**.179**	26.40%	26.00%	**.013**	21.00%	22.91%	**.0001**
IVS‐I‐1(G > A)	1.92%	8.60%	13.30%	7.30%	5.10%	**.08**	9.10%	10.00%	**.105**	4.50%	8.33%	**.461**
IVS‐II‐1(G > A)	1.92%	3.20%	1.10%	2.40%	2.50%	**.921**	1.00%	0%	**.066**	0.60%	0%	**.428**
FSC8(−AA)	0%	9.60%	15.60%	22.00%	13.90%	**.003**	1.00%	1.70%	**.793**	0.20%	0%	**1**
IVS‐I‐6(C > T)	0%	13.90%	14.40%	2.40%	3.20%	**.0001**	6.20%	0.83%	**.017**	0.60%	4.17%	**.078**
IVS‐II‐745(C > T)	0%	0.50%	1.10%	11.00%	7.60%	**.0001**	0%	0%	–	2.60%	4.17%	**.336**
nb of chromosomes	52	187	90	82	158		208	120		475	48	

*Notes*: a: (Lemsaddek et al., 2004), b: (Lemsaddek et al., 2003), c: (Agouti et al., 2007), d: (Agouti, Badens, et al., 2008), e: (Boudrahem‐Addour et al., 2009), f: (Abdaoui et al., 2019), g: (Fattoum et al., 2004), h: (Sahli et al., 2016). Bold indicates *p* value less than .05.

As mentioned above, the C39(C > T) was the most common variant in our cohort but as well as in Western Mediterranean countries and the Maghreb countries (Khelil et al., 2010). The highest frequency was found in Sardinia (95.7%) (Cao et al., 1991) and several studies have suggested a possible Roman origin and support the idea of its introduction into the Maghreb countries during the Roman period and into the western Mediterranean countries by the crusades during the middle ages (Khelil et al., 2010; Zahed, 2001).

The FSC5(‐CT) was the second most prevalent variant in our population (5.77%). This variant has been reported once at homozygous state in Morocco and in Tunisia (Agouti, Badens, et al., 2008; Fattoum et al., 2004). The highest frequency was found in Bulgaria (8.6%) followed by Syria, Lebanon (5%), and Spain (2,7%) (Makhoul et al., 2005; Murad et al., 2018; Petkov & Efremov, 2009; Villegas et al., 2009).

The FSC6(−A) reaches a high frequency in North African countries. It was the third most common variant in Algeria (17.9%–12.9%) and Tunisia (7.02%) and it was the fifth most common variant in Morocco (Agouti, Badens, et al., 2008; Chouk et al., 2004; Labie et al., 1990). It is also frequent in the Portuguese and Bulgarian population (8% and 4.9%, respectively) (Faustino et al., 1999; Petkov & Efremov, 2009).

The IVS‐I‐110(G > A) was well documented as the most common variant in the Eastern Mediterranean countries (Khelil et al., 2010). In North Africa, this variant was reported as the second most common variant in Algeria and Tunisia (26.4% and 21%, respectively) (Boudrahem‐Addour et al., 2009; Fattoum et al., 2004). However, it was rare in Morocco and its frequency varies between 3% and 5%, which is similar to our study (3%). It has been suggested that IVS‐I‐110(G > A) was introduced over the past into Maghreb countries by the Ottoman occupation, which never reach Morocco and the presence of this variant in Morocco could be explained by it recent introduction by migrants from neighboring countries (Khelil et al., 2010; Zahed, 2001).

The −29(A > G) variant was found among black and Asian population and was also reported in Maghreb countries, Spain and China (Pereira et al., 2009; Xu et al., 2004). It was more prevalent in Morocco and less frequent in Algeria and Tunisia (8.5%, 3.8%, and 1.08%, respectively) (Agouti, Badens, et al., 2008; Bennani et al., 1994; Chouk et al., 2004). The frequency decreases from Morocco to Tunisia which could be explained by its introduction by the caravan routes or the expansion of the Almoravid Dynasty (Lemsaddek et al., 2004).

IVS‐I‐1(G > A), IVS‐II‐1(G > A), and −56(G > C) were the rarest variants identified in this study. IVS‐I‐1(G > A) was found among the five most common variants in Maghreb Countries (Agouti et al., 2007; Labie et al., 1990; Lemsaddek et al., 2003). The frequency of this variant was higher in Algeria (10%–14.5%), followed by Morocco (5%–13.3%) and finally, Tunisia (4%) (Abdaoui et al., 2019; Agouti, Badens, et al., 2008; Chouk et al., 2004; Fattoum et al., 2004; Labie et al., 1990; Lemsaddek et al., 2003). Moreover, this variant was also described as the most frequent variant in some European countries such as Czech Republic (27%), Hungary and Middle East countries (Divoka et al., 2016; Ghoti et al., 2017; Ringelhann et al., 1993), and it has been suggested that the variant was originated from Eastern Europe (Makhoul et al., 2005). IVS‐II‐1(G > A) in the other side was the most common variant in Iran with a frequency ranging between 25.61% and 40.33% (Jalilian et al., 2017; Maryami et al., 2015). It was reported as the first or the second most frequent variant in some middle‐Eastern countries and has been found in all Arab countries (Zahed, 2001). This variant has a Mediterranean origin, however, a recent report has hypothesized its West African origin (Broquere et al., 2010). The frequency of this variant in our study is lower compared to that found in previous studies in Morocco (1.96 vs 3.21%–2.53%), but comparable to that in Tunisia (1.62%). In Algeria it was very rare (0.9%) (Agouti, Badens, et al., 2008; Boudrahem‐Addour et al., 2009; Chouk et al., 2004; Lemsaddek et al., 2004).

The −56(G > C) variant was described for the first time in Morocco at compound heterozygous with IVS‐I‐1(G > A) in a woman originated from Algeria. She was diagnosed with β‐thalassemia intermedia and remained asymptomatic without the need of regular transfusion until the age of 18 years old (Agouti, Bennani, et al., 2008). It was also described at homozygous state in a Tunisian patient who had only a mild anemia and never required transfusion (Douzi et al., 2015). In the present study, −56(G > C) was identified in a patient in compound heterozygous with −29(A > G) who had never required transfusion. He showed a typical β‐thalassemia trait hematological features associated with slightly elevated HbF (HBF: 5.8%) and the Complete Blood Count (CBC) was as follows Hb: 10 g/dl, MCV: 60.01 fl, MCHC: 31.6, and HBA2: 5.9%. The patient is stable and takes iron supplement. Therefore, this variant seems to act like a silent variant. Indeed, when combined with a β^0^ variant it can leads to an intermedia phenotype and, when combined with β^+^ variants or in homozygous state it leads to a β‐thalassemia trait. In the heterozygous state, the carriers remain asymptomatic.

Together, these data suggest that the mutational spectrum of β‐thalassemia in eastern region of Morocco is different from that reported in previous studies conducted in Morocco or Algeria and Tunisia. Therefore, each geographic region would have its own type and distribution of variants. This genetic diversity may originate probably gene flow due to population migration. Further studies, based on SNV (Single Nucleotide variation) or STR (Short Tandem Repeat), are necessary to understand the origin of β‐thalassemia variants.

Among the subjects, one was diagnosed with β‐thalassemia minor and Acute Lymphoblastic Leukemia (ALL). He received red cell transfusions combined with iron chelation therapy and chemotherapy. The coexistence of hematological malignancy and β‐thalassemia major is not a rare condition. Multiple cases were reported in different countries with β‐thalassemia major associated with Hodgkin and non‐Hodgkin lymphoma, chronic myeloid leukemia, acute lymphoblastic leukemia, hepatocellular carcinoma, and thyroid malignancies (Alavi et al., 2013; Sherief et al., 2020). However, the association between β‐thalassemia minor and malignancies is very rare.

It has been suggested that there is no relationship between β‐thalassemia and the occurrence of hematological malignancies including ALL. Indeed, the coexistence of β‐thalassemia and the ALL was accidental (Alavi et al., 2013; Sherief et al., 2020) and the ALL could be due to genetic predisposition or others risk factors (Halawi et al., 2017; Tuğcu et al., 2014).

## 5 CONCLUSION

This study provided, for the first time, the molecular investigation of β‐thalassemia in eastern region of Morocco which indicates a high molecular heterogeneity. One variant C39(C > T) accounts for 72%, and the rare variant C38/39(‐C) was described for the first time in Morocco. The mutational spectrum was unique compared to that reported in other region of Morocco or in other North African countries. Therefore, these data are crucial to implement a prevention programs and the establishment of genetic counseling and prenatal detection for β‐thalassemia in Morocco.

## CONFLICT OF INTEREST

The authors declare no conflict of interest.

## AUTHOR CONTRIBUTIONS

*Designing and conception study*: Mohammed Bellaoui, Redouane Boulouiz, and Noufissa Benajiba. *Clinical investigation*: Noufissa Benajiba and Ayad Ghanam. *Molecular and data analysis*: Ihab Belmokhtar, Noufissa Benajiba, Saida Lhousni, Mounia Elidrissi Errahhali, and Manal Elidrissi Errahhali. *Writing and revising manuscript*: Ihab Belmokh, Mohammed Bellaoui, Majida Charif, Meryem Ouarzane, and Redouane Boulouiz.

## INFORMED CONSENT

All the subjects or their representative family gave written informed consent to the study.

## Data Availability

Researchers wishing to use the clinical data and/or biological materials have to complete a BRO Biobank request form. If their request is accepted by the scientific board of the BRO Biobank, a material transfer agreement is signed and the data and biological materials can be supplied.
